# Effects of ferumoxytol on quantitative PET measurements in simultaneous PET/MR whole-body imaging: a pilot study in a baboon model

**DOI:** 10.1186/s40658-015-0109-0

**Published:** 2015-02-26

**Authors:** Ronald JH Borra, Hoon-Sung Cho, Spencer L Bowen, Ulrike Attenberger, Grae Arabasz, Ciprian Catana, Lee Josephson, Bruce R Rosen, Alexander R Guimaraes, Jacob M Hooker

**Affiliations:** Athinoula A. Martinos Center for Biomedical Imaging, Massachusetts General Hospital, 149 13th Street, Suite 2301, Charlestown, MA 02129 USA; Medical Imaging Centre of Southwest Finland, Department of Diagnostic Radiology, University of Turku and Turku University Hospital, Turku, Finland; Center for Advanced Medical Imaging Sciences, Massachusetts General Hospital, Charlestown, MA USA; School of Materials Science and Engineering, Chonnam National University, Gwangju, South Korea; Institute of Clinical Radiology and Nuclear Medicine, University Medical Center Mannheim, Heidelberg University, Mannheim, Germany; Department of Meridian & Acupuncture, Collaborating Center for Traditional Medicine, East-West Medical Research Institute and School of Korean Medicine, Kyung Hee University, Seoul, South Korea; Division of Abdominal Imaging, Department of Radiology, Massachusetts General Hospital, Harvard Medical School, Boston, MA USA

**Keywords:** PET, MRI, PET/MR, Multimodal imaging, Ferumoxytol, Attenuation correction

## Abstract

**Background:**

Simultaneous PET/MR imaging depends on MR-derived attenuation maps (mu-maps) for accurate attenuation correction of PET data. Currently, these maps are derived from gradient-echo-based MR sequences, which are sensitive to susceptibility changes. Iron oxide magnetic nanoparticles have been used in the measurement of blood volume, tumor microvasculature, tumor-associated macrophages, and characterizing lymph nodes. Our aim in this study was to assess whether the susceptibility effects associated with iron oxide nanoparticles can potentially affect measured ^18^F-FDG PET standardized uptake values (SUV) through effects on MR-derived attenuation maps.

**Methods:**

The study protocol was approved by the Institutional Animal Care and Use Committee. Using a Siemens Biograph mMR PET/MR scanner, we evaluated the effects of increasing concentrations of ferumoxytol and ferumoxytol aggregates on MR-derived mu-maps using an agarose phantom. In addition, we performed a baboon experiment evaluating the effects of a single i.v. ferumoxytol dose (10 mg/kg) on the liver, spleen, and pancreas ^18^F-FDG SUV at baseline (ferumoxytol-naïve), within the first hour and at 1, 3, 5, and 11 weeks.

**Results:**

Phantom experiments showed mu-map artifacts starting at ferumoxytol aggregate concentrations of 10 to 20 mg/kg. The *in vivo* baboon data demonstrated a 53% decrease of observed ^18^F-FDG SUV compared to baseline within the first hour in the liver, persisting at least 11 weeks.

**Conclusions:**

A single ferumoxytol dose can affect measured SUV for at least 3 months, which should be taken into account when administrating ferumoxytol in patients needing sequential PET/MR scans.

**Advances in knowledge**

1. Ferumoxytol aggregates, but not ferumoxytol alone, produce significant artifacts in MR-derived attenuation correction maps at approximate clinical dose levels of 10 mg/kg.

2. When performing simultaneous whole-body ^18^F-FDG PET/MR, a single dose of ferumoxytol can result in observed SUV decreases up to 53%, depending on the amount of ferumoxytol aggregates in the studied tissue.

**Implications for patient care**

Administration of a single, clinically relevant, dose of ferumoxytol can potentially result in changes in observed SUV for a prolonged period of time in the setting of simultaneous PET/MR. These potential changes should be considered in particular when administering ferumoxytol to patients with expected future PET/MR studies, as ferumoxytol-induced SUV changes might interfere with therapy assessment.

**Electronic supplementary material:**

The online version of this article (doi:10.1186/s40658-015-0109-0) contains supplementary material, which is available to authorized users.

## Background

The use of fully integrated PET/MR systems as a clinical imaging modality has increased significantly in recent years [1,2]. This novel hybrid technique has many workflow advantages; however, one of the essential prerequisites for its clinical acceptance is that semi-quantitative PET measures such as standardized uptake values (SUV) are not altered by the endogenous administration of intravenous or oral contrast agents such as gadolinium-based contrast agents (GBCA) or iron oxide-based contrast agents.

In order to obtain quantitative PET information, PET/MR imaging relies on MR information that is processed into an MR-derived attenuation map (mu-map), which in turn can be used for attenuation correction (AC) of PET data [3]. This implies that, because both PET and MRI examinations can be performed simultaneously, there is the potential of MR-related temporal changes (such as injected GBCA) to affect the accuracy of PET measurements. A recent study evaluated the possible effects of GBCA on PET accuracy in whole-body PET/MR imaging, and no significant effect was found [4]. However, preliminary data on oral iron oxide-based MRCA in the same study did show a significant effect on the quality of MR-based attenuation correction maps.

Ferumoxytol (Feraheme®, AMAG Pharmaceuticals, Lexington, MA, USA) is an iron oxide-based magnetic nanoparticle and was originally developed as an intravenous MR contrast agent having the benefits of a long intravascular half-life in addition to macrophage avidity secondary to its monocrystalline formulation. Belonging to the class of ultra-small superparamagnetic iron oxides (USPIO), ferumoxytol consists of superparamagnetic iron oxide nanoparticles with a polyglucose sorbitol-carboxymethylether coating [5]. The carbohydrate shell aids to isolate the iron from the plasma components until it is taken up by the reticuloendothelial system of the spleen, bone marrow, and especially liver (up to 70% of injected dose). Ferumoxytol’s structure and iron oxide core result in altered MR contrast on both T1- and T2-weighted imaging by shortening of T1, T2, and T2*. The intravascular half-life of ferumoxytol is long, around 14 to 15 h [6], and as a result, in particular because of storage in tissue, the effects on MR imaging can persist for days up to even months [7]. Its uses as a contrast agent in the initial, post-injection phases include angiography and assessment of tumor microvasculature [8,9], but a second very important reason for the popularity of ferumoxytol as an imaging agent is that it allows for a very reliable, non-invasive assessment of lymph node status in cancer patients, independent of the lymph node size [10]. Ferumoxytol’s use as an MRI contrast agent for clinical indications and its comparison to traditional GBCA have been recently published in the areas of CNS and lymphoma imaging [11-13]. In addition, a very recent study showed that in clinical abdominal imaging, ferumoxytol produces relevant signal changes 48 h after i.v. administration, in particular in the adrenal glands [14]. Phase 3 clinical trials, including subgroups of patients with inflammatory bowel disease that can also potentially greatly benefit from i.v. iron supplementation are currently ongoing to evaluate the use of ferumoxytol in other patient groups with chronic kidney disease (CKD) with iron deficiency anemia (for example, ClinicalTrials.gov identifiers: NCT01114139, NCT01114217, and NCT01114204) [15].

Ferumoxytol (Feraheme® 30 mg/ml, AMAG Pharmaceuticals) was recently approved by the FDA *exclusively* for the intravenous treatment of iron deficiency anemia in patients with CDK [16]. The FDA’s decision is apparently based on the improved safety profile (only a single serious adverse event in 750 CDK patients treated with ferumoxytol) of the drug compared to other iron compounds used to treat anemia in this particular patient group, such as iron dextran [17,18]. Long-term effects caused by ferumoxytol-derived iron accumulation, in particular in the liver and spleen, on abdominal MR data quality have been described in the literature but with very few experiments [19-21]. Average-sized patients are administered a single vial of ferumoxytol, which contains 510 mg of elemental iron. Since the human body on average contains around 4 g of iron, a single dose of ferumoxytol adds a substantial amount of iron to the whole-body iron pool in a very short amount of time.

With the advent of PET/MRI technology, it is unknown whether ferumoxytol will have any deleterious effects on quantification with the attenuation correction algorithms currently used on PET/MRI platforms. In the case of whole-body PET/MR imaging, most commonly, a direct segmentation method is used to generate MR-derived mu-maps, which generally do not account for attenuation by bone tissue [22]. In particular settings where delayed uptake of iron oxide-based agents are used, such as lymph node imaging, this is potentially a significant problem because the workflow does not allow for an MR-based attenuation correction map to be obtained prior to the agent’s administration. The purpose of the current study is to quantify the effects of ferumoxytol on simultaneously obtained quantitative PET/MR data using a phantom and baboon model, in particular possible effects of ferumoxytol over time on observed SUV in multiple abdominal organs. An animal model was chosen for this study because of the need for repeated PET scans within a limited time frame, which would not be possible in human subjects due to the resulting radiation exposure.

We are not aware of any previous studies using ferumoxytol that have addressed this issue. In this study, we use an *in vivo* model to elucidate potential differences in mu-map artifacts caused by ferumoxytol and ferumoxytol aggregates, as we hypothesize that aggregates cause greater artifacts in gradient-echo-based sequences with in turn larger mu-map artifacts as a result.

## Methods

### PET/MR imaging

All simultaneous PET/MR imaging was performed on a Siemens Biograph mMR system (Siemens Medical Solutions U.S.A., Inc., Malvern, PA, USA).

### Phantom study details

An agarose phantom was constructed by filling 310-ml stackable flasks with 1% agarose (Agarose LE, Analytical Grade, Promega Corporation, Madison, WI, USA) and 2, 4, 10, and 20 mg/kg concentrations of ferumoxytol (Feraheme® 30 mg/ml, AMAG Pharmaceuticals, Inc., Lexington, MA, USA) or an identical amount of ferumoxytol aggregated with concanavalin A (Sigma-Aldrich, St. Louis, MO, USA). Large flasks were chosen in order to minimize possible partial volume effects. Ferumoxytol aggregates were produced by adding concanavalin A (Sigma-Aldrich, St. Louis, MO, USA) to ferumoxytol (Feraheme® 30 mg/ml, AMAG Pharmaceuticals, Inc., Lexington, MA, USA). Concanavalin A demonstrates agglutination activity with carbohydrates [23] and formed precipitates with branched polysaccharides [24], which surrounded the bioavailable iron oxide of Feraheme as a protecting layer. Concanavalin A was added to ferumoxytol in a ratio of 30 μM of ConA to 0.7 μg Fe/ml of Feraheme in a buffered (pH 6) solution of 0.15 M NaCl with 1 mM CaCl_2_ and MnCl_2_. Average measured particle size by laser dynamic light scattering (DLS) using a Zetasizer (Malvern Instruments, Marlboro, MA, USA) was 17 to 31 nm (from the prescribing information) before and 848 ± 122 nm after addition of concanavalin A. For each phantom pair (ferumoxytol only vs. ferumoxytol aggregates) of a given concentration, identical procedures were followed and similar amounts of iron oxide-containing solution produced, with the only difference the addition of concanavalin A, in order to optimize comparability and to make sure observed effects were due to aggregates only.

**Figure 1:**
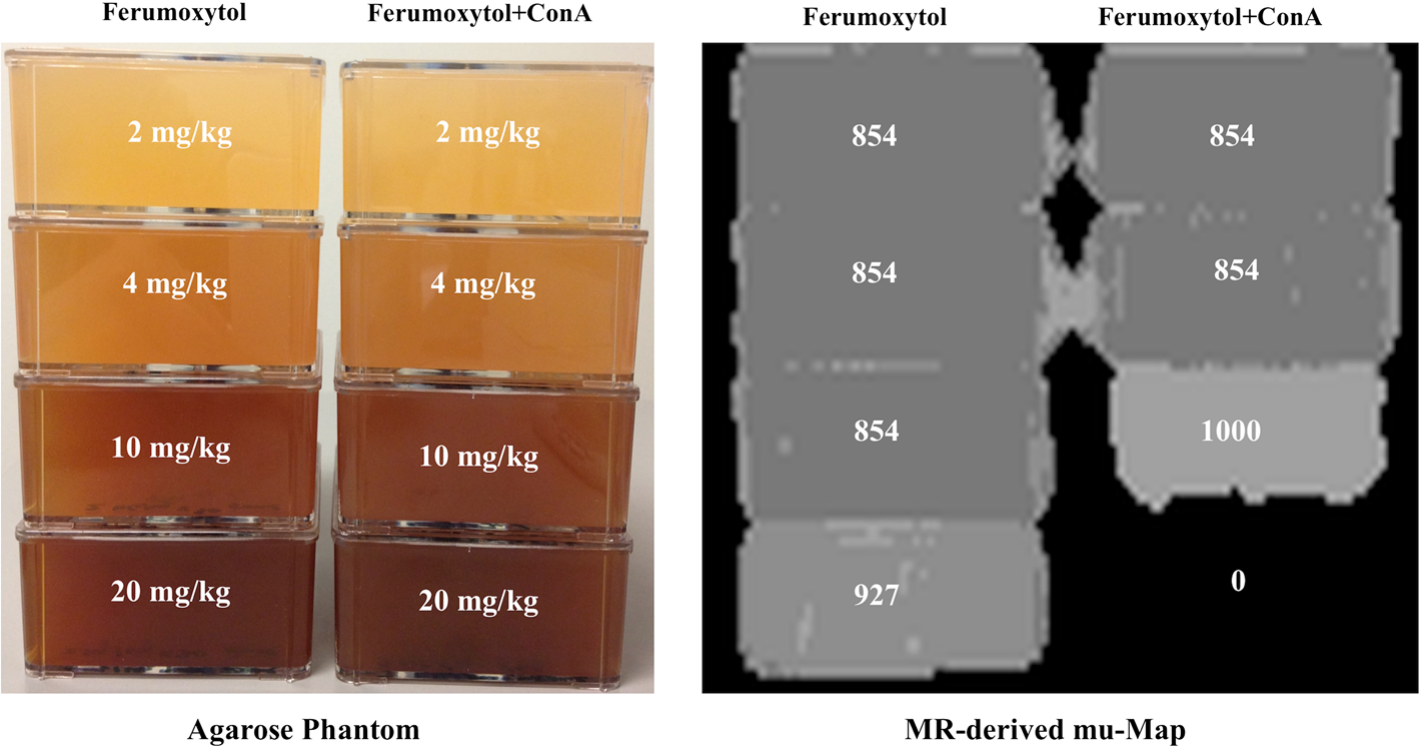
**Phantom studies with ferumoxytol and ferumoxytol aggregates.** Image of an agarose phantom with increasing concentrations of ferumoxytol (left stack) or ferumoxytol concanavalin A (ConA) aggregates (right stack) and the corresponding mu-map. On the VIBE-based MR-derived mu-map, a value of 1,000 reflects soft tissue density and 0 air density. Up to concentrations of 10 mg/kg, the measured value in both phantoms with ferumoxytol and ferumoxytol aggregates was similar, at a value of 854 reflecting fat tissue density. However, significant artifacts in the VIBE-based mu-map due to ferumoxytol aggregates are visible at the concentration of 20 mg/kg, where the content of the agarose phantom is misclassified as air (value of 0) in the VIBE-based mu-map, whereas at the concentration of 10 mg/kg, the observed value is actually higher (1,000) for the ferumoxytol aggregates compared to ferumoxytol alone.

Also, agarose concentration was kept at 1% in all flasks in order not to influence possible differences between concentrations. In order to prevent precipitation of ferumoxytol aggregates, all agarose-filled flasks were rotated during the first 60 min while being rapidly cooled in an ice bath. After the agarose had solidified for 24 h, flasks were imaged using the clinical Siemens Biograph mMR PET/MR system with routinely available sequences. The rationale for the chosen concentrations was to study if tissue ferumoxytol concentrations of a clinical dose of 10 mg/kg (assuming a fully homogeneous distribution in the body) can potentially influence the obtained MR data. A picture of the phantom and corresponding mu-map is shown in Figure 1.

### Animal study details

All procedures in this study complied with the regulations of the Institutional Animal Care and Use Committee of the Massachusetts General Hospital. Baboon studies were performed in the context of other studies performed by our group in order to minimize animal use. A single male baboon (*Papio anubis*) with a mean weight of 13.35 kg (12.7 kg at baseline and 14 kg at 11 weeks) was used for all imaging studies. The animal was fasted at least 12 h prior to the study, and anesthesia was induced with ketamine (20 mg/kg ketamine with 0.4 mg/kg diazepam or 10 mg/kg ketamine with 0.5 mg/kg xylazene). Endotracheal intubation was performed, and a catheter for radiotracer injection was placed in the antecubital vein. Anesthesia was maintained during the PET/MR scan by isoflurane (0.8% to 1.5%, pure oxygen mixture) without active mechanical ventilation. During the scan, physiological parameters (respiratory rate, heart rate, blood pressure, and end-tidal CO_2_) were monitored continuously. The baboon had not been injected with ferumoxytol or other USPIO at least 7.5 months prior to the baseline scan. After the baseline scan (25-min ^18^F-FDG PET during which the described T1W and T2W anatomical sequences were obtained), a single dose of 10 mg/kg ferumoxytol (Feraheme® 30 mg/ml, AMAG Pharmaceuticals, Inc., Lexington, MA, USA) with a volume of 4.23 ml was rapidly injected intravenously, followed by a 10-ml saline flush. In this work, baseline refers to scans acquired on a ferumoxytol-naïve animal, time points in the text and figures to scans acquired after a single administration of ferumoxytol after the baseline scan was performed.

### MR imaging-related details

Imaging was performed using the four-channel Body Matrix coil (Siemens Medical Solutions U.S.A., Inc., Malvern, PA, USA) and the built-in spine coil as the receiving coil elements. Gating was performed using respiratory bellows. The following essential scans for PET/MR data analysis and interpretation were obtained:

- Default MR-based attenuation correction (MRAC) scan: T1-weighted (T1W) two-point Dixon 3D volumetric interpolated breath-hold examination (VIBE) sequence with the following parameters: integrated parallel acquisition technique (iPAT) GRAPPA factor 2, repetition time (TR) 3.6 ms, first echo time (TE1) 1.23 ms, second echo time (TE2) 2.46 ms, matrix size 79 × 192, averages 1, field of view (FOV) 50 cm, flip angle (FA) 10°, slice thickness 5.5 mm, and acquisition time (TA) 0:19 min. The resulting in-phase, out-phase, and fat and water images of the same anatomical location were used as input for the proprietary mu-map reconstruction (including lung compartment) on the scanner. This sequence was used prior to every PET scan and at additional time points to assess the effects of ferumoxytol during the time course of a single PET scan.
- High-resolution anatomical T1W axial, coronal, and sagittal dual-echo scan with the following parameters: TR 115 ms, TE1 1.23 ms, TE2 2.46 ms, matrix size 256 × 168, averages 1, FOV 35 cm, phase FOV 65.6%, FA 32°, slice thickness 4 mm, and approximate TA 0:55 min.
- High-resolution fat-suppressed axial anatomical series T2W turbo spin echo (TSE) with the following parameters: iPAT GRAPPA 2, TR 5,264 ms, TE 98 ms, matrix size 488 × 235, averages 2, FOV 18.0 cm, phase FOV 75%, FA 150°, slice thickness 3 mm, and approximate TA 5:46 min. In addition, as a possible alternative for the VIBE-based MRAC, a two-echo ultra-short echo time (uTE) scan was obtained with the following parameters: TR 11.34 ms, TE1 0.07 ms, TE2 2.46 ms, matrix size 192 × 192, averages 2, FOV 30, phase FOV 100%, FA 10°, slice thickness 1.56 mm, and TA 1:35 min.

### PET imaging-related details

PET data were acquired after the manual i.v. administration of 162.5 ± 2.4 MBq ^18^F-fluoro-2-deoxy-D-glucose (^18^F-FDG) in a 3-ml solution followed by a 10-ml saline flush. Data were obtained dynamically and recorded in list mode format during two (1-, 3-, 5-, and 11-week scans) or three 25-min consecutive PET scans (including the baseline scan in the ferumoxytol-naïve animal), with the first PET scan started at the time of ^18^F-FDG injection and the second and third scans started at 30 and 60 min after tracer injection. PET data were obtained with an axial FOV of 25.8 cm, transaxial FOV of 59.4 cm, and axial and transaxial resolution of ≤4.8 and ≤4.7 mm, respectively. Attenuation correction of raw PET data was performed on the scanner console after the scan using the method provided by the manufacturer based on segmentation of anatomical MR images [22] (see MR imaging-related details below). Data of the second PET scan (30 to 55 min) were reconstructed for each of the time points using a 3D-OSEM method and a 4-mm FWHM Gaussian kernel filter.

### Data reconstruction, correction, and analysis

A single 1,500-s (25 min) static PET dataset was reconstructed for all 30- to 55-min ^18^F-FDG scans for the scans at each time point (baseline, 5 min, 57 min, and 1, 5, and 11 weeks after ferumoxytol injection), and AC was performed using the MRAC scan immediately preceding the acquisition. In addition, for the first visit, the PET data were attenuation corrected with MR-derived mu-maps obtained at 5, 13, 29, 30, 44, and 57 min after the injection of ferumoxytol. Reconstructed and attenuation-corrected PET data and corresponding MR data were exported from the scanner. These files were imported in OsiriX (Osirix Foundation and Pixmeo, Geneva, Switzerland), a software suite equipped with image viewing, fusion, and analysis capabilities [25]. All pixels in PET data were converted to SUV. PET data and corresponding anatomical MR data were automatically fused using Osirix, and fusion quality was visually inspected prior to region of interest (ROI)-based analysis. A visually highly acceptable co-registration of PET/MR datasets was obtained for all measured time points using this method.

**Figure 2:**
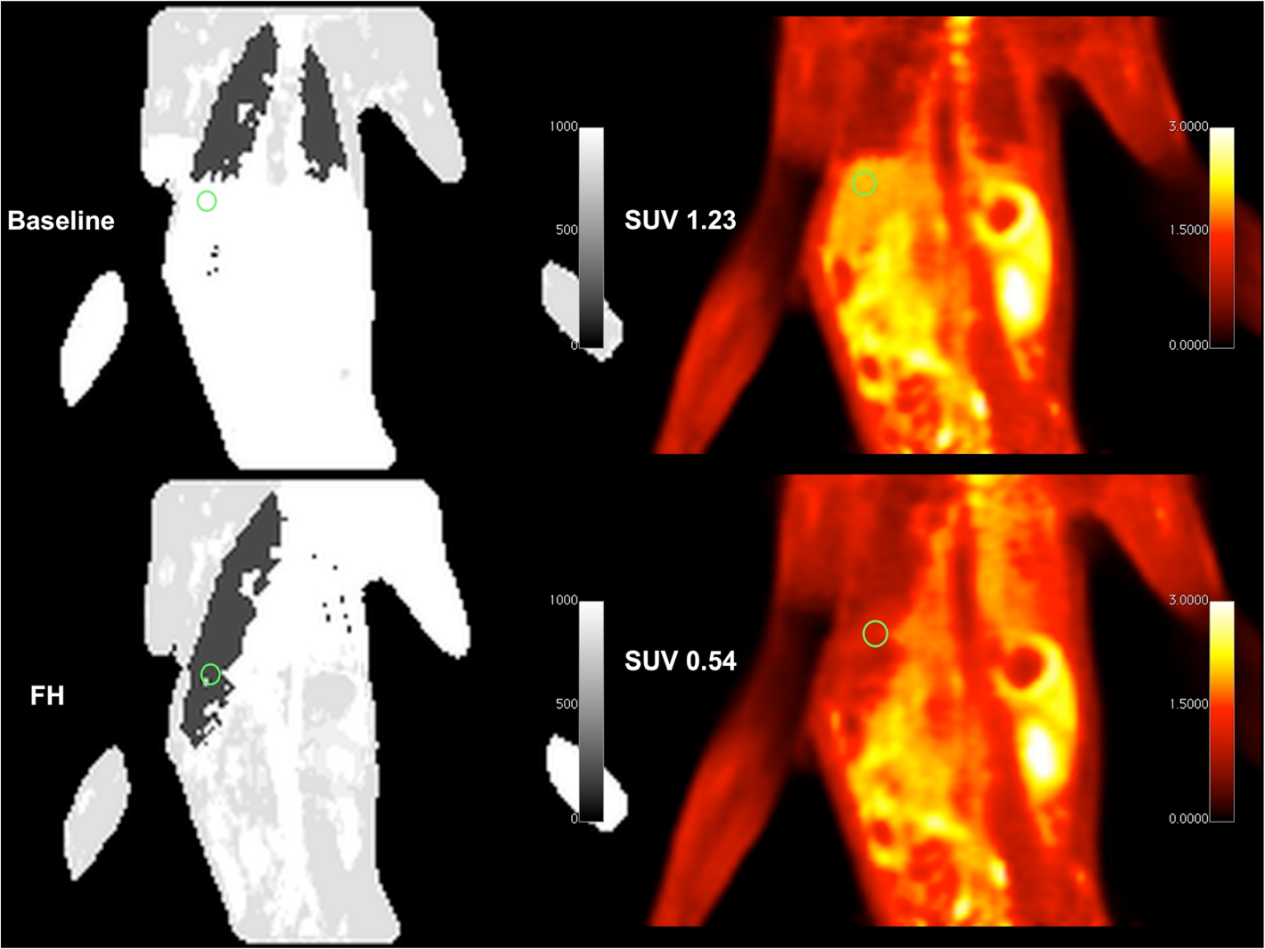
**Effects of ferumoxytol on measured SUV.** Coronal mu-maps (left column) and PET SUV images (right column) showing a typical ROI in the liver region. The top row is data obtained at 5 min after 10 mg/kg ferumoxytol administration (SUV 1.23); the bottom row is data obtained 57 min after ferumoxytol administration (SUV 0.535). PET raw data is identical in both instances; however, attenuation correction has been performed with the mu-map on the left of each PET dataset. Please note the significant liver area misclassification at the 57-min time point (signal drop due to high liver iron content).

Liver SUV was measured using a single two-dimensional (2D) circular ROI (size 1.464 cm^2^) drawn using coronal T1W images. In order to obtain as identical as possible ROI placement between scan time points, coronal MR data were viewed side by side and corresponding levels and locations within the liver were visually identified through anatomical landmarks in the coronal series and available orthogonal T1W MR series, which were acquired using real-time respiratory gating in order to minimize motion-induced artifacts within and between series. Drawn ROIs were then propagated to co-registered parametric ^18^F-FDG PET SUV images. In the case of the spleen and pancreas, an oval ROI (size 0.999 and 0.248 cm^2^, respectively) was drawn on high-resolution T2W TSE images in the center of the spleen and the corpus area of the pancreas, and then propagated to the PET SUV datasets, similarly to the liver measurements. Mean SUV values were recorded for each ROI. Typical location of the liver ROI and effect of incorrect AC on PET SUV are shown in Figure 2.

## Results

### Phantom imaging

Phantom imaging results are displayed in Figure 1. Because of signal loss due to ferumoxytol aggregates (at 20 mg/kg), the VIBE-based MR-derived mu-map is affected and indicates a different density, although the true density (agarose) is identical. The average observed linear attenuation coefficient (LAC) values with a unit of cm^−1^ (here multiplied by 1,000) are indicated for each flask. No significant artifacts were observed at ferumoxytol and ferumoxytol aggregate concentrations up to 10 mg/kg and below (classified as fat, value of 854) with minor differences in mu-map values at 10 mg/kg (which could be partially due to the proximity of the flask to the very high dose 20 mg/kg aggregate flask) with the phantom with 10 mg/kg aggregates being incorrectly classified as soft tissue (value of 1,000). However, very clear differences at 20 mg/kg concentrations for both the ferumoxytol and ferumoxytol aggregate were observed with the ferumoxytol flask incorrectly classified as a mix between fat and soft tissue (value of 927) and the ferumoxytol aggregate flask incorrectly classified as air (value of 0).

### Longitudinal imaging in a baboon before and after i.v. ferumoxytol administration

**Figure 3:**
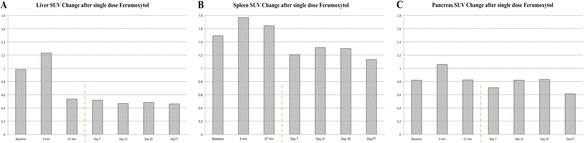
**Short- and long-term effects of ferumoxytol on SUV.** Overview of ^18^F-FDG SUV changes in the liver **(A)**, spleen **(B)**, and pancreas **(C)** before and after 10 mg/kg i.v. ferumoxytol administration.

Mu-maps obtained within the first 5 min of ferumoxytol injection resulted in changes to the mu-map that corresponds to AC PET images with increased SUV. All mu-maps obtained at >13 min demonstrated significant signal loss in the liver, leading to misclassification of the liver tissue as lung tissue. This is illustrated in Figure 2 with the mu-maps and corresponding AC PET images for time points 5 and 57 min after injection of ferumoxytol. Effects of ferumoxytol at these timepoints on the individual VIBE images are illustrated in Additional file 1. Errors in mu-maps resulted in significant changes in observed SUV, particularly in the liver, both in the acute stage (within 1 h) and at later time points after injection of ferumoxytol. SUV data for the liver, spleen, and pancreas are displayed in Figure 3.

In the case of the liver, in the acute stage, a maximum SUV drop versus baseline was observed at 44 min after injection (0.98 vs. 0.46), reflecting a decrease of 53%. Interestingly, after 1 h, the observed SUV stayed fairly constant up to the 11-week time point. A similar trend involving smaller absolute changes was observed in the case of the spleen and pancreas (Figure 3). The biggest change in SUV measured in the spleen area vs. baseline was at the last time point (11 weeks, 77 days), with a decrease of 24% (1.49 vs 1.13) in observed SUV. A similar observation was noted in the case of the pancreas (0.81 vs 0.61, 25% decrease).

**Figure 4:**
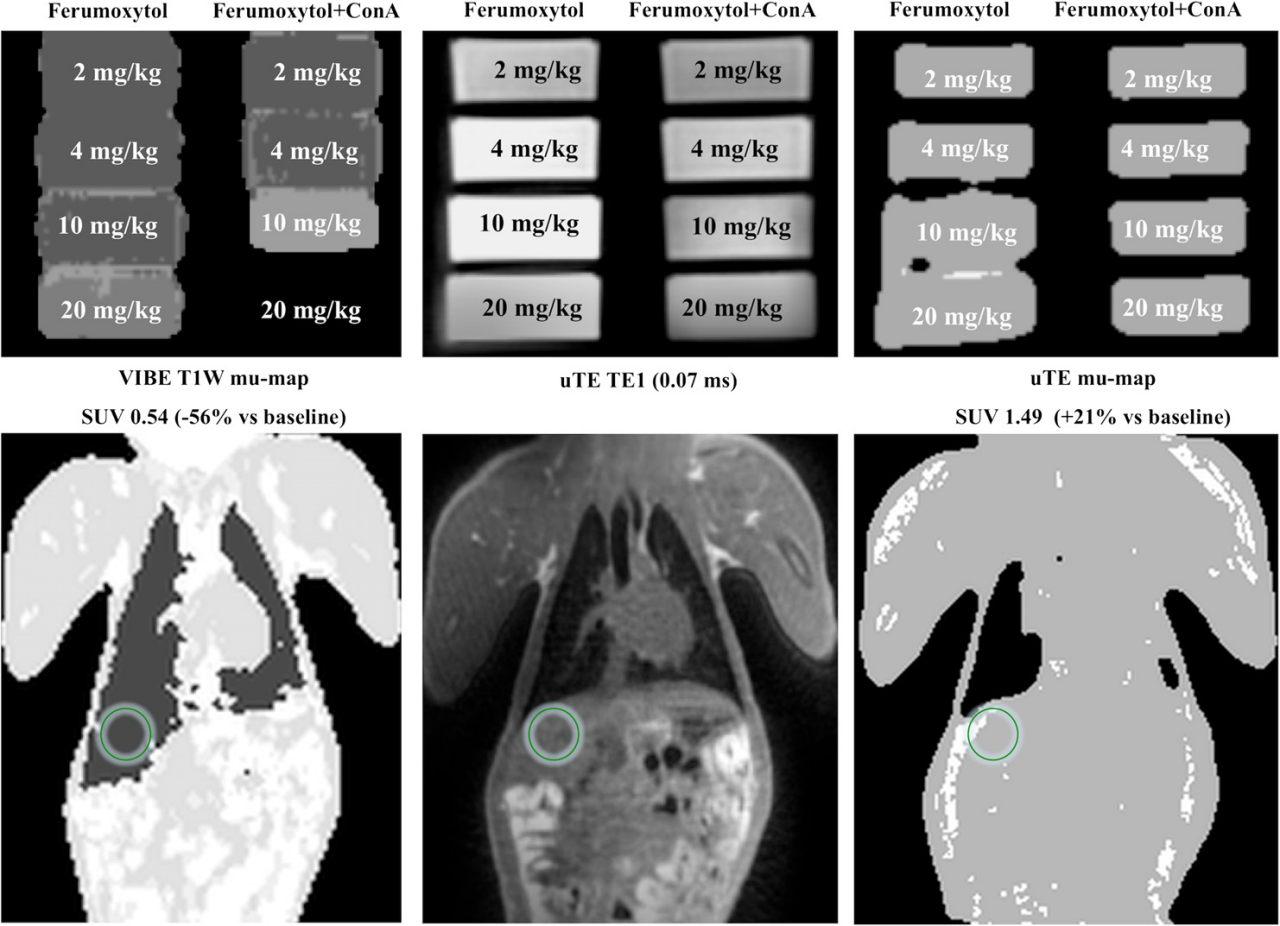
**Ultra-short echo-time imaging after ferumoxytol administration.** Example of how ultra-short echo-time imaging can allow for detection of tissue anatomy even in the presence of high tissue iron content. The top row again reflects the same phantom as in Figure 1, imaged with the VIBE-based sequence (left panel). The middle panel shows the same phantom but now imaged with an ultra-short TE-based sequence, with the resulting anatomical image fairly unaffected even by high amounts of ferumoxytol aggregates. The right panel shows the mu-map derived from the uTE-based MR data. Below each phantom dataset is an example of anatomical data that can be obtained *in vivo* with the same sequence. It can be observed that in the left panel, the mu-map is incorrect due to high liver iron content, causing the liver to be classified as lung when using the VIBE-based mu-map. However, due to the very short TE, the uTE sequence is able to detect signal in the liver in the same scan which in turn is reflected by high signal in the uTE-derived mu-map. However, it can be observed that the uTE-derived mu-map lacks detail elsewhere in the body (for example, the tissue of the left lung is incorrectly segmented) and therefore requires further optimization for this purpose. The mu-map quality in the liver is reflected by the SUV values measured from the corresponding PET datasets (data not shown) with the uTE-based mu-map causing a slight overestimation of the observed SUV compared to baseline (+21%); however, this difference is much smaller than the underestimation due to the iron-induced artifacts in the VIBE-based mu-maps (−53%).

Data obtained with the uTE-based sequence in both the ferumoxytol phantom and the baboon are displayed in Figure 4. The uTE sequence allowed for the detection of MR signal in both phantom and *in vivo* even in the presence of high tissue iron content, with as a result an improved MR-derived mu-map in tissues with significant iron accumulation.

## Discussion

The results of the current study suggest that, depending on the studied organ, a single dose of ferumoxytol can potentially change observed SUV by as much as 53% when using the standard VIBE-based MR-derived mu-maps for PET data attenuation correction. Initially (approximately the first 5 min), during and just after the bolus injection of ferumoxytol, a change in VIBE signal leads to an increased calculated SUV for PET data - the longer term observed effect of ferumoxytol-induced changes in VIBE images, which persisted for months, and the resulting negative SUV changes in a healthy baboon were strongest in the liver and much less predominant in the spleen and pancreas.

To support the difference in early versus late signal changes observed after ferumoxytol administration, we compared phantoms prepared with graded concentrations of either free ferumoxytol or iron aggregates formed from ferumoxytol. Indeed, the highest concentration of free ferumoxytol led to an increase in the derived mu-map value (LAC), whereas the highest concentration of ferumoxytol aggregates dramatically decreased the mu-map value. These data are consistent with the mechanism proposed for signal differences in the NHP experiments.

Although ferumoxytol is currently not FDA approved as an MR contrast agent, the reports in the current literature show that its off-label use has increased significantly over the past years and numerous research studies are currently ongoing to further understand the benefit of ferumoxytol in cancer and tumor microenvironment imaging. Taking into account the aforementioned increased popularity of the drug, fuelled by its unique ability to assist with non-invasive lymph node staging in cancer imaging, the main reasons why we believe our current findings are of potential (future) clinical importance are twofold:

First, our data strongly suggest that the current method for MR-derived attenuation correction in simultaneous whole-body PET/MR is not optimized to deal with high tissue iron concentrations, and therefore, the decision to administer ferumoxytol to a patient (in a research or other setting) should be carefully considered, especially in case future PET/MR scans are considered, for example, for the purpose of treatment evaluation. Second, our data suggests that, despite the major acute effects (within hours), the long-term effects of ferumoxytol (several months) are fairly constant over time. This potentially provides an opportunity of comparing PET studies with MR-derived AC in the time frame where little (further) change due to tissue iron accumulation can be expected.

Although the findings presented in this manuscript were obtained in a single baboon, we feel that this work points out a highly interesting interaction between the MR and PET modality that warrants further investigation. For example, the findings would need to be reproduced in a larger group, and also possible measures to prevent or reduce the effect altogether, such as (early) minimally invasive chelation therapy, should be explored.

In addition to highlighting the scarce knowledge with regard to the detailed pharmacokinetics of ferumoxytol in different organs and tissues *in vivo*, the current work also provides technical perspectives, in particular visualization of the liver using a uTE-based sequence despite high levels of iron, that might aid future methods to obtain artifact-free MR-derived mu-maps. An alternative potentially elegant solution, which was recently proposed by others, might be an optimized combination of both VIBE/Dixon and uTE MR data of the same anatomical location in order to produce robust and high-quality mu-maps [26]. The injected ferumoxytol dose in the baboon of 10 mg/kg, although on the higher end of a clinical dose for iron replacement therapy and an optimal dose for MR brain CBV studies [12], is likely high for abdominal imaging, especially in the case of the liver where the blood volume (≈15%) is approximately three times that of the brain (≈5%). This might have exacerbated the effect of ferumoxytol on SUV that we observed.

## Conclusions

The use of ferumoxytol, although currently not approved as a routine MR contrast agent, is likely to grow due to its highly interesting vascular, macrophage, and in particular lymph node activity. However, based on data presented in this manuscript, we conclude that in the context of simultaneous PET/MR, improved dose response studies and in particular improved MR-derived AC algorithms are needed prior to its utilization for cancer staging.

## Additional file

Additional file 1:
**Effect of ferumoxytol injection on individual VIBE images and the resulting mu-map.**The effects of ferumoxytol injection on the individual VIBE images (in-phase, out-phase, fat and water) are different at 5 min than at 57 min after injection, which in turn results in different mu-maps.
