# Supplementary material for: Effects of ferumoxytol on quantitative PET measurements in simultaneous PET/MR whole-body imaging: a pilot study in a baboon model
Source: EJNMMI Phys. 2015 Feb 26;2:6. doi: 10.1186/s40658-015-0109-0 (PMC4544618; doi:10.1186/s40658-015-0109-0)
Supplement: Additional file 1: — Effect of ferumoxytol injection on individual VIBE images and the resulting mu-map.The effects of ferumoxytol injection on the individual VIBE images (in-phase, out-phase, fat and water) are different at 5 min than at 57 min after injection, which in turn results in different mu-maps. [file 40658_2015_109_MOESM1_ESM.pdf]

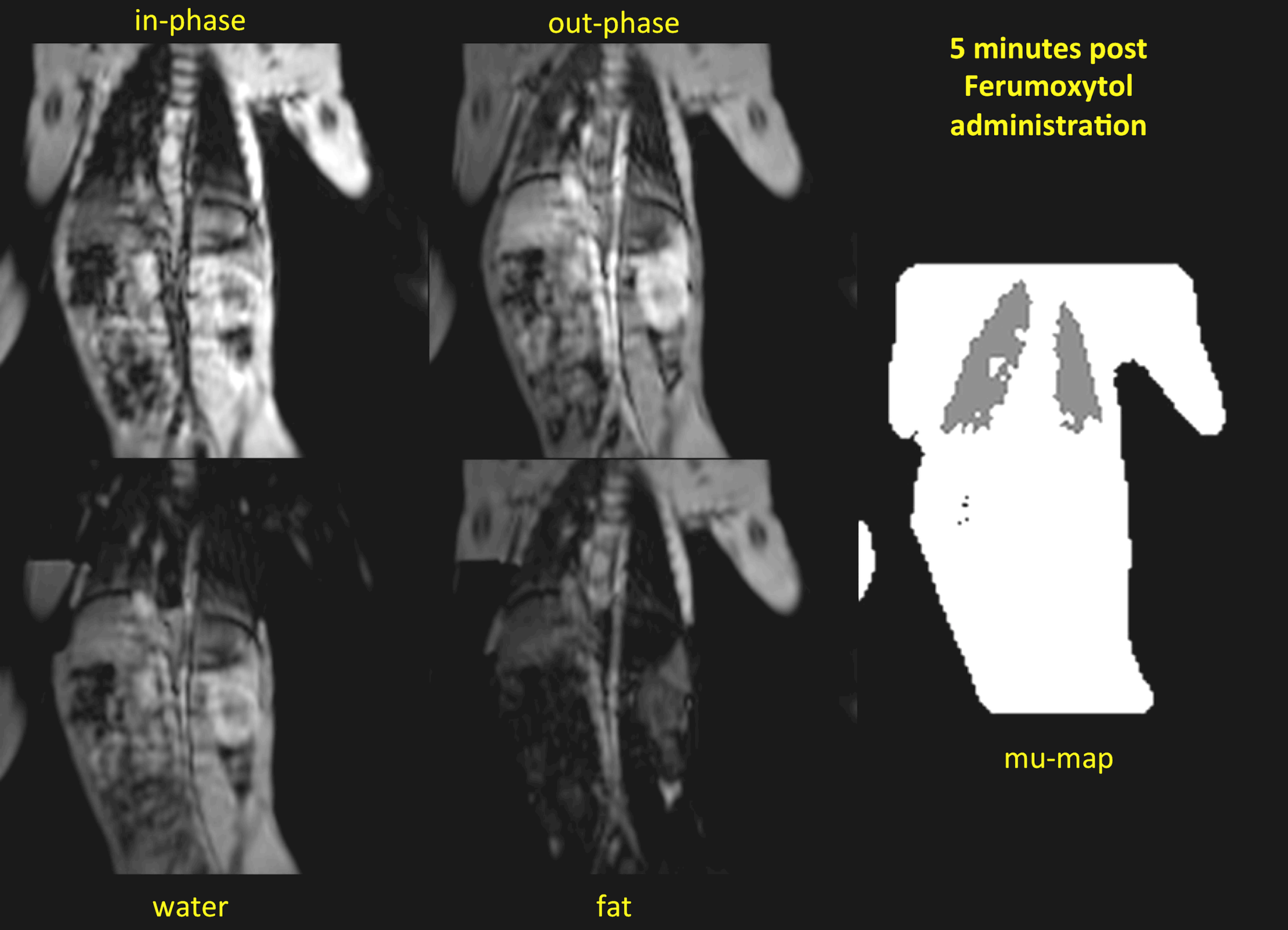
in-phase
out-phase
5 minutes post
Ferumoxytol
administration
water
fat
mu-map

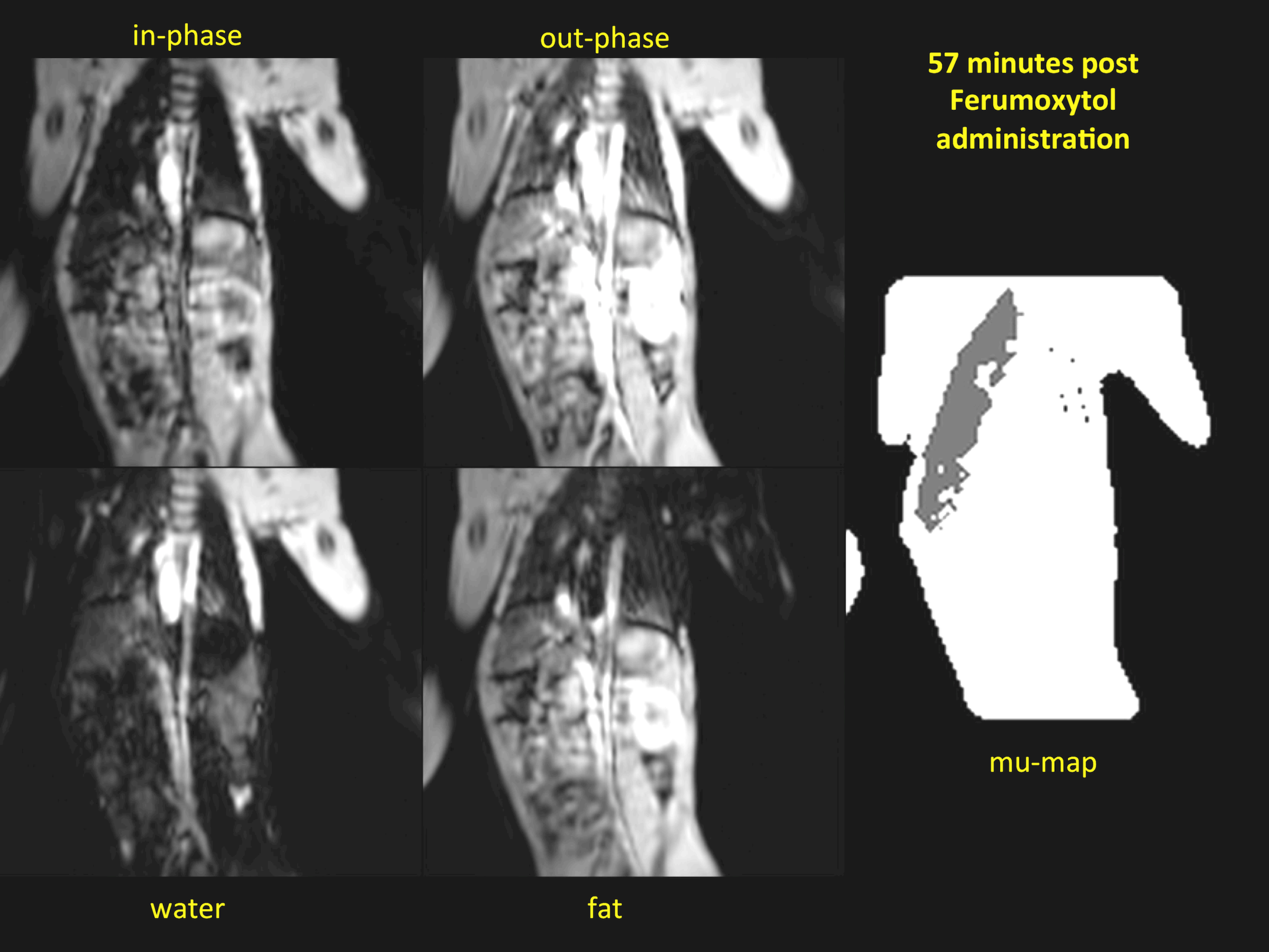
in-phase
out-phase
57 minutes post Ferumoxytol administration
mu-map
water
fat
